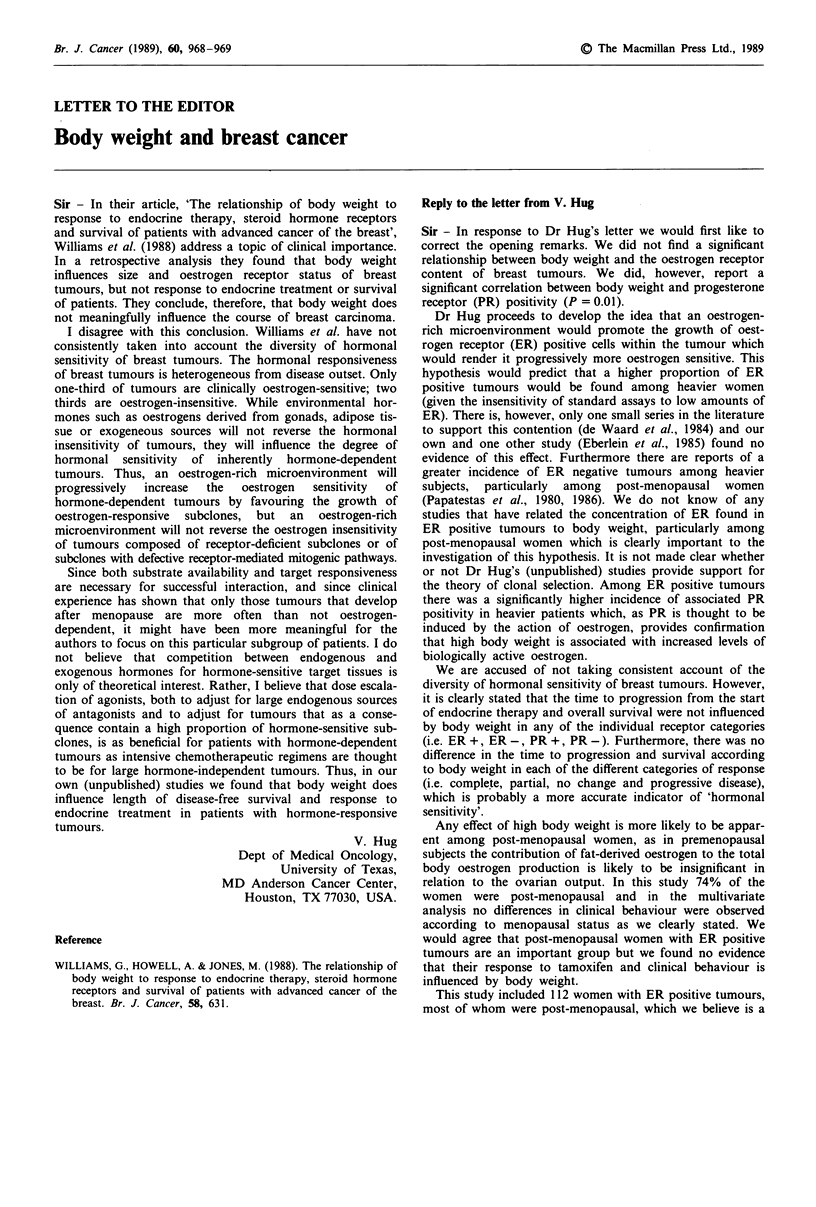# Body weight and breast cancer.

**DOI:** 10.1038/bjc.1989.400

**Published:** 1989-12

**Authors:** V. Hug


Br. J. Cancer (1989), 60, 968–969 © The Macmillan Press Ltd., 1989

### LETTER TO THE EDITOR

### Body weight and breast cancer

**Sir** – In their article, 'The relationship of body weight to response to endocrine therapy, steroid hormone receptors and survival of patients with advanced cancer of the breast', Williams *et al.* (1988) address a topic of clinical importance. In a retrospective analysis they found that body weight influences size and oestrogen receptor status of breast tumours, but not response to endocrine treatment or survival of patients. They conclude, therefore, that body weight does not meaningfully influence the course of breast carcinoma.

I disagree with this conclusion. Williams *et al.* have not consistently taken into account the diversity of hormonal sensitivity of breast tumours. The hormonal responsiveness of breast tumours is heterogeneous from disease outset. Only one-third of tumours are clinically oestrogen-sensitive; two thirds are oestrogen-insensitive. While environmental hormones such as oestrogens derived from gonads, adipose tissue or exogeneous sources will not reverse the hormonal insensitivity of tumours, they will influence the degree of hormonal sensitivity of inherently hormone-dependent tumours. Thus, an oestrogen-rich microenvironment will progressively increase the oestrogen sensitivity of hormone-dependent tumours by favouring the growth of oestrogen-responsive subclones, but an oestrogen-rich microenvironment will not reverse the oestrogen insensitivity of tumours composed of receptor-deficient subclones or of subclones with defective receptor-mediated mitogenic pathways.

Since both substrate availability and target responsiveness are necessary for successful interaction, and since clinical experience has shown that only those tumours that develop after menopause are more often than not oestrogen-dependent, it might have been more meaningful for the authors to focus on this particular subgroup of patients. I do not believe that competition between endogenous and exogenous hormones for hormone-sensitive target tissues is only of theoretical interest. Rather, I believe that dose escalation of agonists, both to adjust for large endogenous sources of antagonists and to adjust for tumours that as a consequence contain a high proportion of hormone-sensitive subclones, is as beneficial for patients with hormone-dependent tumours as intensive chemotherapeutic regimens are thought to be for large hormone-independent tumours. Thus, in our own (unpublished) studies we found that body weight does influence length of disease-free survival and response to endocrine treatment in patients with hormone-responsive tumours.

V. Hug
Dept of Medical Oncology,
University of Texas,
MD Anderson Cancer Center,
Houston, TX 77030, USA.

**Reference**

WILLIAMS, G., HOWELL, A. & JONES, M. (1988). The relationship of body weight to response to endocrine therapy, steroid hormone receptors and survival of patients with advanced cancer of the breast. *Br. J. Cancer*, **58**, 631.